# Holding All the CARDs: How MALT1 Controls CARMA/CARD-Dependent Signaling

**DOI:** 10.3389/fimmu.2018.01927

**Published:** 2018-08-30

**Authors:** Mélanie Juilland, Margot Thome

**Affiliations:** Department of Biochemistry, University of Lausanne, Epalinges, Switzerland

**Keywords:** paracaspase, RNA stability, ubiquitin, Treg, BCR, TCR, GPCR, EGFR

## Abstract

The scaffold proteins CARMA1-3 (encoded by the genes *CARD11, -14* and -*10*) and CARD9 play major roles in signaling downstream of receptors with immunoreceptor tyrosine activation motifs (ITAMs), G-protein coupled receptors (GPCR) and receptor tyrosine kinases (RTK). These receptors trigger the formation of oligomeric CARMA/CARD-BCL10-MALT1 (CBM) complexes via kinases of the PKC family. The CBM in turn regulates gene expression by the activation of NF-κB and AP-1 transcription factors and controls transcript stability. The paracaspase MALT1 is the only CBM component having an enzymatic (proteolytic) activity and has therefore recently gained attention as a potential drug target. Here we review recent advances in the understanding of the molecular function of the protease MALT1 and summarize how MALT1 scaffold and protease function contribute to the transmission of CBM signals. Finally, we will highlight how dysregulation of MALT1 function can cause pathologies such as immunodeficiency, autoimmunity, psoriasis, and cancer.

## Introduction

The past few years have seen considerable advances in our understanding of the role of MALT1 in different biological processes. It is now well accepted that MALT1 acts downstream of various receptors in a complex composed of CARMA (CARD-containing MAGUK) proteins, the adaptor protein BCL10 and MALT1 itself. These so-called CBM complexes are crucial for the regulation of the classical NF-κB pathway (also called canonical or NF-κB1 pathway) and other biological processes that will be covered in this review. We will first introduce the receptors that signal via CBM complexes and then describe the role of MALT1 in CBM-dependent signal transmission and its role in the activation of a variety of downstream signals. Finally, we will highlight how dysfunction of MALT1 can cause pathological alterations, which may be amenable to treatment with MALT1 inhibitors that are presently under development.

## Various receptors signal via CARMA/CARD proteins and MALT1

**Figure 1:**
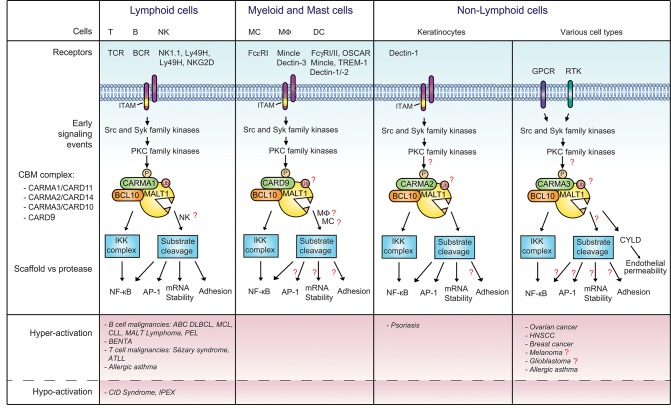
Signaling via MALT1 downstream of various receptors and CARMA/CARD proteins. CBM complexes composed of MALT1, BCL10 and the indicated CARMA/CARD proteins are formed downstream of receptors containing immunoreceptor tyrosine-based activation motifs (ITAMs), G-protein-coupled receptors (GPCRs) or receptor tyrosine kinases (RTKs). Once formed, the CBM complex activates downstream signaling events via the scaffold and protease function of MALT1. The CBM complex of T and B cells is best characterized, while CBM complexes in other cellular contexts are less well studied. Open questions that remain to be addressed are annotated with a red question mark. Deregulated activation of the pathway results in different diseases, as summarized in the lower panel of the figure. BCR, B cell receptor; DC, dendritic cell; EGFR, epidermal growth factor receptor; FcR, Fc receptor; Mφ, macrophage; MC, myeloid cell; NK, natural killer; OSCAR, osteoclast associated receptor; PKC, protein kinase C; TCR, T cell receptor; TREM-1, triggering expression by myeloid cells 1; RA, rheumatoid arthritis.

MALT1 is a ubiquitously expressed protease that resides in the cytoplasm as a catalytically inactive BCL10-bound proenzyme. It is recruited into CBM signaling complexes upon the stimulation of specific receptors, namely those containing one or more immunoreceptor tyrosine-based activation motifs (ITAMs), G-protein-coupled receptors (GPCRs), or receptor tyrosine kinases (RTKs) (Figure 1) (1).

Various immunoreceptors of the innate and adaptive immune response activate MALT1. A common feature of such receptors is the presence of one or more ITAMs, which is crucial for transmembrane signal transmission (2). The ITAM can be present in the cytoplasmic tail of the immunoreceptors, as is the case for the zymosan receptor Dectin-1 present on dendritic cells (DC), or the FcεRI on mast cells. Alternatively, the ITAM can be present in a receptor-associated signaling subunit. For example, in CD3 chains associated with the T-cell receptor (TCR), the CD79 chains associated with the B-cell receptor (BCR) and the FcRγ chain associating with the myeloid cell receptor Dectin-2 or the natural killer (NK) cell receptors NK1.1/Ly49D/Ly49H/NKG2D (2, 3). Upon receptor triggering, Src family kinases phosphorylate the ITAMs, thereby providing a docking site for Syk family kinases. Once recruited, Syk kinases induce the downstream signaling events, which converge toward the activation of PKC family kinases, PKC-dependent phosphorylation of the CARMA/CARD protein and the subsequent formation of a CBM complex, for which the composition is specific for different receptor types (Figure 1).

A common feature of all receptors signaling via CBM complexes is the use of MALT1 as a scaffold protein for the IKK-dependent activation of the heterodimeric transcription factors of the NF-κB1 subtype, p50-RelA and p50-cRel (referred to as NF-κB). As a scaffold protein, MALT1 binds the ubiquitin ligase TRAF6, which subsequently modifies itself, BCL10, and the IKK subunit NEMO by K63-linked polyubiquitin chains (4–7). These modifications are required for the downstream activation of the IKK complex (4, 6), which phosphorylates the NF-κB inhibitor, IκBα, to trigger its proteasomal degradation (8, 9). The protease activity of MALT1 makes an additional contribution to the intensity and persistence of the NF-κB response by cleaving the proteins A20 and RelB, which act as negative regulators of NF-κB activation (10, 11). However, whether all CBM-dependent signaling pathways rely on the protease activity of MALT1, or whether some receptors only rely on the MALT1 scaffold activity remains poorly understood.

The relevance of MALT1 protease activity is strongly evidenced for ITAM-containing receptors, which promote activation of lymphoid and NK cells via CARMA1/CARD11 (11–13) and myeloid cells via CARD9 (13, 14). The protease activity of MALT1 also plays a role outside the immune system, in the activation of keratinocytes (15). Stimulation of keratinocytes with zymosan, which triggers the ITAM-containing receptor Dectin-1, leads to CBM complex activation and MALT1 protease activity via CARMA2/CARD14. This suggests that all ITAM containing receptors signal via both the scaffold and protease activity of MALT1 in immune and non-immune cells.

A variety of GPCRs (such as AT1R, Par-1, PAFR, ET_A_, ET_B_, CXCR2, CXCR4, LPA1-6) and RTKs (the EGFR and HER2/neu) have been shown to trigger the formation of a CBM complex comprising CARMA3/CARD10 (Figure 1) [see (1) and references therein]. Whether the protease function of MALT1 is involved or not in the transmission of the signaling pathway of these receptors is not well understood. For one GPCR (the thrombin receptor PAR1), the protease activity of MALT1 was found to be crucial for the regulation of the integrity of epithelial barriers, whereby stimulation of epithelial cells with thrombin lead to MALT1 proteolytic activity and CYLD cleavage (16), in addition to the previously described formation of a CBM complex comprising CARMA3 (17). These findings suggest the possibility of a broader involvement of the protease activity of MALT1 in CBM-dependent GPCR signaling. In contrast, signaling via the human EGFR2 (HER2), a receptor tyrosine kinase (RTK), has been proposed to depend mainly on the scaffold activity of MALT1 (18). It is possible that MALT1 cleaves a distinct subset of substrates in epithelial cells, or that MALT1 activation requires co-stimulation of the EGFR and another, yet to be identified co-receptor. A need for co-stimulation is observed in T cells, where optimal CBM complex assembly and activation of MALT1 protease activity requires the synergistic stimulation of both, the TCR and the costimulatory receptor CD28 (11, 12, 19, 20). Whether MALT1 protease activity is activated downstream of RTK merits further exploration.

## Multiple elements control the assembly and architecture of the CBM complex

**Figure 2:**
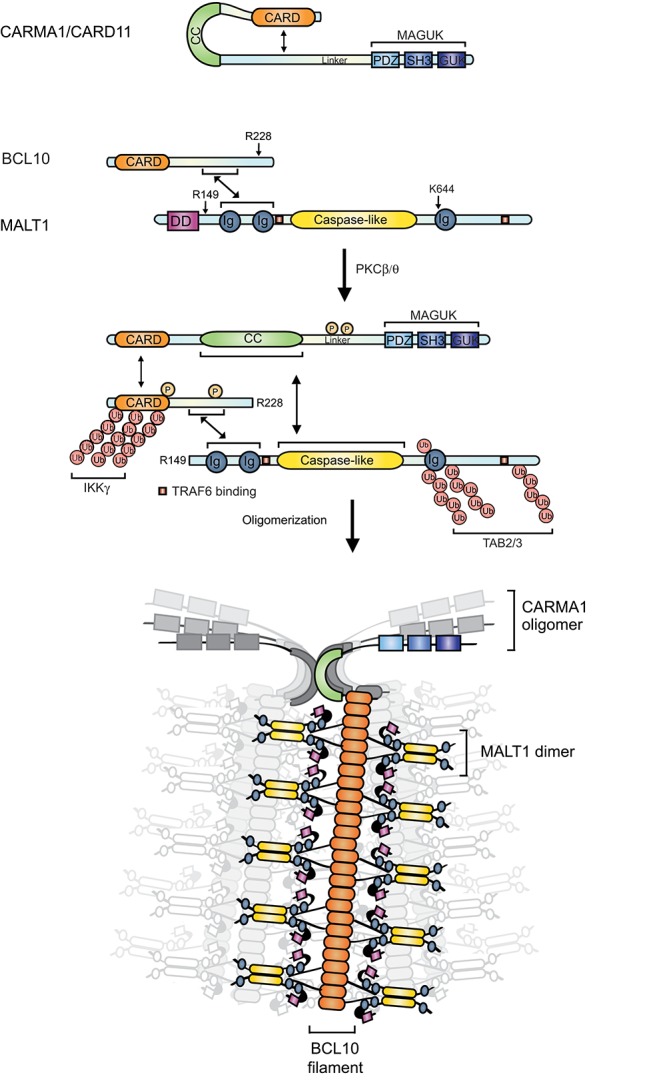
Structure and function of the CBM complex. In resting conditions, MALT1 and BCL10 interact constitutively, while CARMA1 is dissociated from the other components of the CBM. PKC-mediated phosphorylation triggers a conformational opening and oligomerization of CARMA1, leading to the recruitment of preformed MALT1- BCL10 complexes. The E3 ligase TRAF6 is further recruited and polyubiquitinates MALT1 and BCL10. This favors the recruitment of the IKK and TAB2/3-TAK1 complexes and the downstream activation of NF-κB and AP-1. N-terminal phosphorylation of BCL10 by IKKβ enhances NF-κB activation, while a C-terminal phosphorylation disassembles MALT1 from BCL10. BCL10 can undergo both, linear polyubiquitination and K63-linked polyubiquitination, which are important for IKKγ/NEMO recruitment. Monoubiquitination activates MALT1 protease function. Once activated, MALT1 cleaves several substrates including BCL10 (after R228) and itself (after R149). The CBM signal is thought to be amplified by formation of filamentous BCL10 structures initiated by CARMA1 nucleation. BCL10 filaments are decorated with MALT1, most likely in its dimeric form. Double-headed arrows indicate interactions. Arrows indicate P, phosphorylations; Ub, ubiquitination.

A common feature of receptors that signal via MALT1 is their capacity to induce the formation of a specific type of CBM signaling complex. In resting cells, the members of the CBM complex reside in an inactive form in the cytoplasm. The catalytically inactive proenzyme MALT1 binds constitutively to BCL10, while the CARMA/CARD scaffold proteins are kept in a monomeric inactive form due to intramolecular inhibitory interactions. The inducible assembly of the CBM complex requires specific protein domains present in each CBM component (Figure 2). The CARD proteins CARMA1 (CARD11), CARMA2 (CARD14), and CARMA3 (CARD10) are structurally similar. Each contains an N-terminal CARD, followed by a coil-coiled (CC) domain and a C-terminal region that shares features with proteins of the membrane-associated guanylate kinase (MAGUK) family, which contain a PDZ (PSD95, Dlg1, ZO-1), SH3 (Src homology 3), and GUK (guanylate kinase) domain. CARD9 contains a CARD and coiled-coil motif but lacks the C-terminal MAGUK features (21–25). An important structural feature of BCL10 is its N-terminal CARD that allows its inducible association with a CARMA/CARD component (21–25). Additionally, BCL10 contains a C-terminal region with a short sequence of amino acids (107-118) that is necessary for its constitutive interaction with MALT1 (21, 26). MALT1 contains an N-terminal death domain (DD) of unknown function, followed by two immunoglobulin (Ig)-like domains required for BCL10 binding, a caspase-like protease domain and a third Ig domain that plays a regulatory role for the protease function (27, 28) (Figure 2).

**Figure 3:**
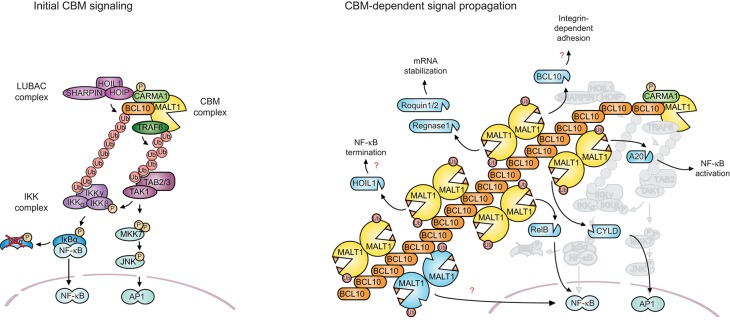
MALT1 scaffold and protease functions. **(A)** Antigen receptor triggering leads to the formation of the CBM complex that acts as a recruiting platform for the ubiquitin ligase TRAF6 and the linear ubiquitin chain assembly complex (LUBAC). TRAF6-dependent K63-linked polyubiquitination promotes IKK complex activation by recruiting TAB2/3 together with TAK1 into proximity of the IKK complex. The TAB2/3-TAK1 complex also promotes AP-1 signaling through MKK7 and JNK activation. The CBM complex additionally recruits the LUBAC complex via a CARMA1-HOIP interaction. This is thought to support NF-κB signaling through linear ubiquitination of BCL10, which promotes the physical recruitment of IKKγ/NEMO. **(B)** Once CBM complex formation has initiated the early signaling events, the signal is most likely amplified and propagated by BCL10 oligomerization and filament formation, and through the protease function of MALT1. Filaments might support the protease function of MALT1 by promoting its dimerization and monoubiquitination. Filaments might also favor or control access of active MALT1 to cellular substrates. The protease activity of MALT1 controls NF-κB activation, AP-1 activation, mRNA stability, and cellular adhesion. This is mediated by the cleavage of the depicted substrates (depicted in blue, also for cleaved BCL10 and autoprocessed MALT1).

The inducible assembly of the CBM complexes seems to depend on a common, conserved mechanism that requires the receptor-induced activation of PKC family members (29, 30). These Ser/Thr kinases (and likely other kinases) phosphorylate the CARMA/CARD proteins in the linker region (Figure 2, illustrated here for CARMA1 (CARD11)) (29–31). This in turn is thought to weaken an inhibitory intra-molecular interaction between the CARD and linker domain, thereby promoting a CARD-mediated association with BCL10 and the formation of the CBM complex (29–31). Similar mechanisms are thought to control activation of CARMA3 (26), CARMA2 (15), and CARD9 (32), but whether PKC family members directly phosphorylate CARMA2 or CARMA3 is not known. CBM complex formation depends on homotypic CARD-CARD interactions between the CARMA/CARD proteins and BCL10 (21–23, 25, 26) and possibly on an additional interaction of the protease domain of MALT1 with the CC domain of CARD proteins as was demonstrated for CARMA1 (33). The CC region of CARMA1 has been shown to mediate oligomerization that is required for TCR-induced NF-κB activation (30, 34). Structural architecture studies and modeling suggest that the CC domain-mediated oligomerization of CARMA1 nucleates the oligomerization of BCL10 into helical filaments. BCL10 filaments are formed in a cooperative manner and stabilized through homotypic interactions formed between the CARD motifs of adjacent BCL10 monomers in the filament (35). MALT1 is constitutively associated with BCL10, and therefore forms part of these filamentous structures (Figures 2,3) (35). Although not filamentous in nature, oligomeric signaling complexes containing proteins with CARD motifs, such as the apoptosome, inflammasome, and pyddosome, have been proposed to be key to the activation of proteases of the caspase family (36). Therefore, a prime purpose of the formation of BCL10-MALT1 filaments is likely to allow the oligomerization-dependent activation of MALT1. Crystallographic data from several groups support the idea that active MALT1 forms a dimer that is stabilized by interactions between the protease domain (37, 38). It is tempting to speculate that BCL10 filaments form a scaffold that permits additional interactions between MALT1 dimers leading to its proteolytic activation (Figure 2). MALT1 activation strictly requires monoubiquitination in its Ig3 domain that promotes its activation in an intra-or intermolecular manner (39). Exactly how this monoubiquitination contributes to MALT1 activation within the context of the oligomeric BCL10-MALT1 fibers remains unexplored. Although this has not yet been formally addressed, it is likely that similar oligomerization-dependent protein complexes control CBM-driven signaling for all CARMA/CARD proteins.

## MALT1 function is determined by its multidomain structure

The *MALT1* gene was originally identified as the target of recurrent translocations in MALT lymphomas (40–42). Later on, *in silico* studies uncovered that MALT1 shares homology with caspases and metacaspases and therefore falls into a category of caspase-like proteases coined paracaspases (27, 43). The partial structure of the MALT1 caspase-like domain together with the third Ig domain has been recently solved and confirms the expected structural similarities between the protease domains of MALT1 and caspases (37, 38).

Apart from sharing structural homology, several other aspects of MALT1 function are shared with caspases, including cysteine-dependent protease activity (11, 12) a requirement for dimerization (38, 44), and the capacity to auto-process (45, 46). Like caspases, MALT1 contains a protease domain with a catalytic dyad comprising two amino acids that are essential for catalysis, namely C464 and H415 (27). Monomeric MALT1 is inactive and, as for caspases, requires dimerization via the caspase-like protease domain for enzymatic activity (38, 44, 47) (Figure 3B). Interestingly, MALT1 has been recently shown to undergo autoprocessing. However, unlike caspases, which autoprocess within the protease domain, MALT1 cleaves itself between the N-terminal death domain and the first Ig domain (45, 46). This does not seem to enhance proteolytic activity but is essential for MALT1-dependent NF-κB activation by a mechanism that is not yet understood (45, 46). In contrast to caspases, the proportion of cellular MALT1 undergoing autoprocessing is very small. It is unclear whether this reflects activation of only a very small proportion of cellular MALT1 or a subtler regulation of its activity, which needs to be investigated in more detail.

Despite the above-mentioned structural similarities between caspases and MALT1, several important differences exist. One major difference between caspases and MALT1 resides in their substrate specificity. While caspases are aspartic acid-specific proteases, MALT1 cleaves its substrates after arginine residues (11, 12, 47). The consensus cleavage site of MALT1 based on known substrates and screening of a tetrapeptide library, is a peptide sequence composed of an arginine at the P1 position, which is in most cases followed by a G or A residue. A hydrophobic residue is usually present at position P4, a rather variable range of amino acids is tolerated at P3 and a preference for serine or proline was found for position P2 (47). The consensus cleavage site could thus be summarized as follows: Φ-X-P/S-**R**'-G/A.

A second important difference between caspases and MALT1 concerns the regulation of MALT1 function, which is dependent on posttranslational modification (PTM) by ubiquitination. MALT1 is ubiquitinated, upon antigen receptor triggering, within the Ig3 domain and its C-terminal part by either K63-linked polyubiquitination or monoubquitination. MALT1 polyubiquitination mediated by TRAF6 is crucial for the activation and/or physical recruitment of the IKK complex, which phosphorylates the NF-κB inhibitor IκB to initiate its degradation and thus allows the nuclear translocation of NF-κB (4, 6, 7). This K63-linked polyubiquitination of MALT1 likely provides docking sites for the adaptor protein TAB2, which forms a complex with the IKK-activating kinase TAK1 (48). Additionally, the K63-linked ubiquitin chains may serve to physically recruit the IKK complex via its catalytically inactive IKKγ subunit (4, 49). MALT1 mono-ubiquitination on lysine residue 644 (K644), on the other hand, is required for the activation of MALT1 protease activity, most likely by favoring the formation of an active dimeric form of MALT (39).

A third aspect that differentiates MALT1 from caspases is the specific presence of additional protein domains that support its regulation and function. In the N-terminal part preceding the protease domain, MALT1 contains an N-terminal death domain (DD) followed by two Ig-like domains. The C-terminal part following the caspase-like domain contains a third Ig-like domain and a structurally undefined (most likely flexible) C-terminal extension (Figure 2). DDs can serve as protein-protein interaction domains in apoptotic signaling pathways. However, and despite its structural similarity with caspases, MALT1 does not induce apoptosis. This suggests that the MALT1 DD recruits another, yet to be identified, DD-containing protein unrelated to apoptosis. It is also possible that this domain plays an inhibitory role, since MALT1 autoprocessing, which removes the DD, is required for optimal NF-κB activation (45, 46). Additional unique features of MALT1 are the presence of three Ig domains that contribute to protein-protein interactions, and the presence of TRAF6 binding motifs. The first two Ig domains of MALT1 are necessary for the binding to BCL10 and are therefore crucial for its ability to induce CBM-mediated NF-κB activation upon engagement of the TCR (24). The region between the second Ig domain and the caspase-like domain contains a motif that is important for TRAF6 binding (4, 5), which is lacking in a recently described MALT1 splice variant (see below) (50) (Figure 2). Additional TRAF6 binding sites are present in the C-terminal extension, which are important for the downstream signaling event (4). The third, C-terminal Ig domain interacts physically with the protease domain and has a particularly important role in MALT1 protease activity, which is regulated by mono-ubiquitination (Figure 2) (39). This and other posttranslational modifications of MALT1 and the CBM complex are important for downstream signaling events that will be discussed below.

## The CBM complex forms a scaffolding platform for protein recruitment

Once assembled, the CBM complex acts as a signaling platform for the recruitment of multiple signaling complexes, including TRAF6, the TAB1/2-TAK1 complex, the IKK complex, and the linear ubiquitin chain assembly complex (LUBAC) (51). These control various aspects of signal transmission that are essential for NF-κB and AP-1 activation.

The activation of NF-κB requires both the physical recruitment of the IKK complex and its activation by the Ser/Thr kinase TAK1. Recent data suggest an important role for both TRAF6 and the LUBAC complex in promoting IKK recruitment (4, 52). The LUBAC complex is composed of three subunits known as HOIL1 (heme-oxidized IRP2 ubiquitin ligase 1), HOIP (HOIL1-interacting protein; also known as RNF31), and SHARPIN (SHANK-associated RH domain interacting protein). The HOIP subunit has an E3 ligase activity, while HOIP and SHARPIN are important for LUBAC complex regulation, such as assuring its proper localization or stability (52, 53). The CBM complex can associate with the LUBAC complex in a transient manner in stimulated T cells, with kinetics that are similar to the association of LUBAC with the IKK complex (54). The stimulation-induced phosphorylation and conformational opening of CARMA1 is thought to lead to recruitment of both, BCL10 and HOIP (55). BCL10 thereby becomes accessible for HOIP-mediated linear ubiquitination on K17, K31, and K63 residues (55). The linear ubiquitination of BCL10 may promote NF-κB activation by providing docking sites for the physical recruitment of NEMO/IKKγ via its UBAN domain (55). However, alternative mechanisms may contribute to IKK recruitment, since another study showed a role for the LUBAC complex in optimal NF-κB activation that was independent of HOIP's ubiquitin ligase activity (54). Indeed, K31 and K63 on BCL10 can be subject to both linear or K63-linked polyubiquitination (7, 55) and the BCL10 modification by K63-linked ubiquitin chains may also contribute, although with lower affinity, to the physical recruitment of NEMO/IKKγ (7, 49, 56).

The CBM-associated E3 ubiquitin ligase responsible for synthesis of K63-linked ubiquitin chains is TRAF6, which associates with oligomeric BCL10-MALT1 complexes (4) and can be physically incorporated into the BCL10-MALT1 filaments in a cooperative manner (57). It has therefore been proposed that BCL10-MALT1 oligomers serve to activate TRAF6 to promote the ubiquitination-dependent activation of the IKK complex (4) (Figure 3). MALT1 is polyubiquitinated by TRAF6 on multiple sites preceding the protease domain and within the Ig3 domain (Figure 2). These polyubiquitination events are critical for NF-κB activation, since mutation of key amino acids important for TRAF6 binding to MALT1 impairs TCR-induced NF-κB activation (4, 5, 50). The TRAF6-mediated polyubiquitination of MALT1 and/or itself is thought to recruit the TAB2/3-TAK1 complex (4), since the adaptor proteins TAB2/3 contain ubiquitin-binding motifs that bind to K63 polyubiquitin chains (58). TAK1, which associates with TAB2/3, is thereby brought into proximity of the IKK complex to phosphorylate the IKKβ subunit. IKKβ phosphorylation activates the IKK complex, which phosphorylates the NF-κB inhibitor IκB, inducing its degradation to allow nuclear entry of NF-κB (4). In parallel, TAK1 contributes to AP-1 activation via the MKK7-JNK pathway (Figure 3) (59).

Interestingly, a recent study suggests a novel regulatory mechanism for MALT1-dependent TRAF6 recruitment by alternative splicing of MALT1 (50). In primary T cells, two MALT1 isoforms can be expressed, namely MALT1A or MALT1B, the latter of which lacks 11 amino acids encoded by exon 7 (50). These 11 amino acids encode for the first TRAF6 binding site, localized between the second Ig like domain and the paracaspase domain of MALT1 (5, 50) (Figure 2). Resting primary T cells express MALT1B, while MALT1A expression is induced upon TCR triggering (50). Therefore, TCR-induced expression of MALT1A likely reinforces the TRAF6-recruiting scaffolding function of the CBM complex (50).

Collectively, these findings support the notion that formation of the CBM complex has a key role in signal transmission as a protein recruitment scaffold.

## The CBM complex promotes MALT1 proteolytic activity

The above-described steps of protein recruitment downstream of the CBM complex are most likely early signaling events that occur within the first minutes of lymphocyte stimulation, since peak IκB phosphorylation is detectable within a few minutes after antigen receptor engagement. The scaffolding function of MALT1 may then be amplified and propagated by its assembly with the BCL10 filamentous structures that are nucleated by CARMA1, as depicted in Figures 2, 3. The activation of the MALT1 protease activity most likely depends on further amplification of the signal through filament formation, since optimal MALT1 activity is detectable later than IKK activation, and peaks roughly 30 min after antigen receptor engagement (11, 12, 19, 20). How exactly filament formation could contribute to MALT1 activation remains unknown. Interestingly, monoubiquitination on K644 within the Ig3 domain of MALT1 is required to license MALT1 protease activity (39). Indeed, a point mutation of K644 preventing monoubiquitination of MALT1 has strongly reduced protease activity and an impaired capacity to sustain IL-2 production in activated T cells (39). The incorporation of MALT1 into filaments might promote MALT1 activation in various ways, for example by allowing recruitment of the (unknown) ubiquitin ligase responsible for MALT1 monoubiquitination and/or assuring physical proximity between MALT1 and its substrates (Figure 3).

## MALT1 cleaves various substrates with diverse biological consequences

The last 10 years have witnessed impressive progress in the understanding of MALT1 protease function and its role in NF-κB- dependent and -independent aspects of lymphocyte activation. The best-explored function of MALT1 protease activity is its contribution to NF-κB activation. While MALT1 scaffold-dependent IKK activation promotes rapid but transient NF-κB activation, long-lasting NF-κB activation is assured by the MALT1-dependent cleavage of negative regulators of NF-κB, including RelB (10) and A20 (11), together with MALT1 autoprocessing (45, 46) (Figure 3). RelB plays a critical role as an inhibitor of the canonical NF-κB pathway in lymphocytes, by forming transcriptionally inactive complexes with RelA and c-Rel (10). MALT1-dependent RelB cleavage results in RelB proteasomal degradation, thereby favoring RelA-, and c-Rel-dependent NF-κB activation (10). MALT1-dependent cleavage of the deubiquitinating enzyme (DUB) A20, on the other hand, relieves its inhibitory role in NF-κB activation (11). How exactly A20 cleavage promotes NF-κB activation remains incompletely understood. It has been proposed that A20 negatively regulates the scaffolding function of MALT1 by removing the MALT1 polyubiquitination (60), however this function of A20 does not seem to be affected by its cleavage, since MALT1 inhibitors do not affect IKK-mediated NF-κB activation. A third cleavage event that is important for NF-κB activation is MALT1 autoprocessing (45, 46). MALT1 is autoprocessed after R149, between the N-terminal DD and the first Ig domain. This cleavage is essential for NF-κB activation downstream of its nuclear accumulation by mechanisms that are not yet elucidated (Figure 2) (45). Finally, NF-κB can be negatively regulated by the recently identified MALT1 substrate HOIL1, which is part of the LUBAC complex (61, 62). HOIL1 cleavage by MALT1 diminishes linear ubiquitination of cellular proteins, and has thus been proposed to provide negative feedback on the NF-κB pathway (61, 62).

Another important transcriptional pathway that is regulated by MALT1 is the JNK/AP-1 pathway. While the scaffold function of MALT1 is required for JNK activation (63), the protease activity of MALT1 provides an additional layer of control, via the cleavage of the deubiquinating enzyme (DUB) CYLD (64). CYLD cleavage is unlikely to affect JNK activation, since MALT1 knock-in mice showed no defect in inducible JNK phosphorylation (13, 14, 65, 66), Thus, CYLD cleavage regulates AP-1 through another mechanism that remains to be explored (Figure 3). Despite the described role of CYLD in the negative regulation of NF-κB and the fact that CYLD shares certain substrates with A20 (such as TRAF2, TRAF6, RIP1, and IKKγ) (67), CYLD cleavage is not implicated in TCR-dependent canonical NF-κB activation (64).

Several studies have identified additional NF-κB- and AP-1 independent roles for MALT1. One of these roles is the control of the stability of particular subsets of mRNA transcripts. The MALT1-dependent cleavage of the RNAse Regnase-1 (also known as MCPIP-1 or Zc3h12a) (68) and of the two RNA binding proteins Roquin-1 and Roquin-2 (69) has been shown to stabilize mRNAs by distinct mechanisms. Regnase-1 is an RNAse that cleaves stem loop structures in the 3′ untranslated region (UTR) of mRNAs, including the mRNAs of Regnase-1 itself, c-Rel, ICOS, OX40, IL-2, and IL6 (70). T cell specific Regnase-1 knockout mice developed spontaneous inflammation and premature death, demonstrating the crucial role of Regnase-1 in the regulation of mRNA stability during the adaptive immune response (68). Roquin-1 and Roquin-2 proteins bind to mRNAs via their ROQ domain, which recognizes a secondary RNA structure called constitutive decay element (CDE), and regulate gene expression at the posttranscriptional level (71). Roquin-1 and its paralog Roquin-2 are functionally interchangeable (72). To degrade mRNAs, Roquins recruit an mRNA deadenylase complex via their C-terminal part (71) and regulate the half-life of key cytokines, chemokines, and costimulatory proteins such as ICOS, OX40, and TNF (71, 73, 74). Similar to Regnase-1 deficient mice, Roquin mutant mice (expressing a Roquin mutant that cannot bind CDEs), develop autoimmunity (73). Regnase-1 and Roquins regulate mRNA decay of common and Regnase-1- or Roquin-specific mRNAs. During T cell triggering, the combined mRNAse activity of Regnase-1 and the mRNA decay activity of Roquins are overcome by their MALT1-dependent cleavage and degradation (Figure 3) (68, 69).

Finally, the protease activity of MALT1 also regulates transcription-independent cellular functions by the cleavage of BCL10, which promotes lymphocyte adhesion through β1 integrins (11, 12, 19, 20). MALT1-mediated BCL10 cleavage removes 5 amino acids from its C-terminus (11, 12, 19, 20). This cleavage is not required for antigen-dependent NF-κB activation but is important for integrin-mediated T-cell adhesion (11, 12, 19, 20). The molecular mechanisms controlling adhesion via BCL10 remain elusive (Figures 2, 3).

Thus far, 8 different substrates of MALT1 have been functionally described in activated lymphocytes, and two additional substrates (NIK and LIMA1) have been reported as specific substrates of an oncogenic cIAP2-MALT1 fusion protein specifically expressed in MALT lymphoma (75, 76). We can therefore reasonably anticipate that MALT1 has additional substrates with functions that are much wider than anticipated. For example, MALT1 protease function has been suggested to play a key role in metabolic pathways (77). Glutamine uptake is rapidly initiated upon naive T cell activation via the amino acid transporter ASCT2 and this uptake is crucial for activation of the mammalian target of rapamycin complex 1 (mTORc1). Interestingly, both glutamine uptake and mTORc1 activation required proteins of the CBM complex and MALT1 protease activity (77). This suggests a novel role for the MALT1 protease function in the regulation of glutamine uptake via the regulation of mTORc1 and/or ASCT2 (77). Whether this regulation is mediated by one of the already described substrates of MALT1, or whether a yet unidentified MALT1 substrate can modulate the mTORc1 signaling pathway remains to be explored.

## Mice lacking MALT1 expression or activity have distinct immunological phenotypes

The protease activity and most of the substrates of MALT1 have been initially identified and characterized in cell lines. However, recent studies using mouse models have lent strong support to the physiological relevance of MALT1 protease activity in adaptive immunity. MALT1-deficient mice show impaired B- and T-cell responses together with defects in the development of particular lymphocyte subsets such as marginal zone (MZ) and B1 B cells (63, 78). Moreover, these mice are resistant to experimental induction of experimental autoimmune encephalitis, a mouse model of multiple sclerosis (79, 80). Mice expressing a catalytically inactive mutant of MALT1 [C472A knock-in mice, subsequently called MALT1-protease inactive (PI) mice] recapitulate these aspects of the immunodeficiency phenotype of the MALT1 knockout mice to a large extent. Lymphocytes isolated from the MALT1-PI mice also revealed defects in NF-κB activation, AP-1 activation, mRNA stability and lymphocyte adhesion that are all consistent with previous studies from cell lines (13, 14, 65, 66, 81). Despite these impaired responses, the lymphocytes isolated from MALT1-PI mice showed normal antigen receptor-mediated activation of IKK or JNK (63, 78). The impaired cellular responses and the associated immunodeficiency phenotype of the MALT1-PI mice results thus principally from the inactivation of the proteolytic activity of MALT1.

In addition to defects in T- and B-cell responses, MALT1-deficient mice have impaired myeloid and mast cell responses upon Fc-receptor stimulation, reduced NK cell responses and defective innate immunity to yeast infections (63, 78, 82–84). The cellular responses of myeloid and NK cells have also been assessed in MALT1-PI mice and found to be defective.

A surprising finding of all four studies reporting MALT1-PI mice, however, is that these mice develop autoimmune symptoms, in particular a severe autoimmune gastritis of early onset, which progresses with age (13, 65, 66). The observed autoimmunity is likely the result of reduced development of natural Treg (nTreg) cells in MALT1-PI mice (13, 65, 66). Indeed, the autoimmune phenotype of MALT-PI mice can be rescued through transfer of wildtype Tregs into newborn MALT1-PI animals (13, 65). An even stronger reduction of nTregs is found in MALT1-deficient mice (13, 65, 66). However, MALT1-deficient mice show no autoimmune pathology, most likely because their lymphocyte responses are more dramatically affected (13, 14, 63, 65, 66). The reduced Treg numbers are likely caused by the absence of MALT1 function during development, since no spontaneous autoimmunity develops in adult mice treated with small molecule MALT1 inhibitors for up to 17 days (85). Indeed, young MALT1-PI mice do not show autoimmune features and are completely resistant to induction of experimental autoimmune encephalitis (13, 65). While the contribution of individual MALT1 substrate(s) to nTreg development remains largely unknown, a recent study suggests that MALT1 autoprocessing is important for nTreg development (46). Indeed, mice expressing an autoprocessing-resistant mutant of MALT1 have a partial Treg deficit that results in enhanced anti-tumor immunity without causing autoimmunity (46).

Mice deficient for the individual CARMA/CARD family members have additional phenotypic features, including a defect in innate immune responses against bacteria, fungi or viruses in CARD9 KO mice (82, 86, 87) and a specific defect in NF-κB signaling upon stimulation of GPCRs in CARMA3-deficient mice (88). Whether these phenotypes depend on MALT1 protease activity remains largely unexplored. In addition, roughly half of CARMA3- and one third of BCL10-deficient mice die prematurely due to a neuronal tube defect, suggesting a common role for CARMA3 and BCL10 in embryonic development (88, 89). This defect is neither present in MALT1-deficient nor in MALT-PI mice and therefore likely depends on MALT1-independent signaling features of CARMA3 and BCL10. CARMA2/CARD14-deficient mice have been recently generated and found to be resistant to psoriasis induction by imiquimod cream or recombinant IL-23 injection (90). The individual contributions of keratinocytes and γδ T cells to the CARMA2/CARD14 dependent-inflammation and the extent to which MALT1 protease activity contributes to inflammatory skin reactions remain to be further investigated.

## MALT1 deregulation is associated with immunodeficiency and lymphoma

**Figure 4:**
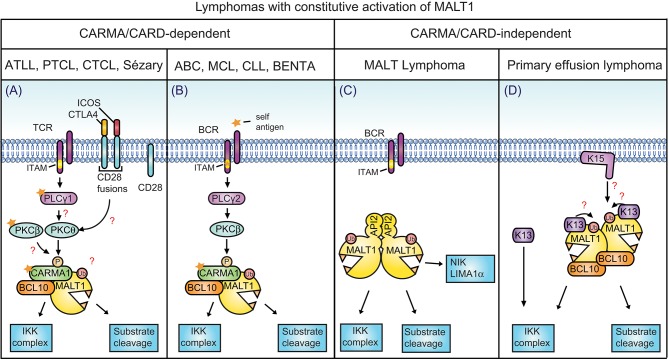
CARMA/CARD-dependent and-independent MALT1 activation. **(A)** In the depicted T cell malignancies, gain-of-function mutations in CARMA1 or its upstream regulators or in-frame gene fusions of the T-cell co-receptor CD28 with ICOS or CTLA-4 are thought to promote MALT1 constitutive activation. **(B)** In the indicated B-cell lymphomas constitutive MALT1 protease activity can be driven by oncogenic mutations in CARMA1, CD79A or CD79B, which may be relevant in combination with self-antigen recognition. A subset of MCL and CLL are also addicted to this pathway by unknown mechanisms. **(C)** A large proportion of MALT lymphoma harbors a chromosomal translocation leading to formation of an oncogenic MALT1-API2 fusion protein. **(D)** In primary effusion lymphoma (PEL), NF-κB is activated through viral latency proteins such as K13 and K15, which activate NF-κB directly or via MALT1-BCL10. **(A,B)**, mutations and self-antigen are depicted with a yellow star. **(A,D)** Open questions are depicted with red question marks. PTCL, peripheral T-cell lymphoma; ATLL adult T-cell leukemia/lymphoma; ABC, activated B-cell subtype; DLBCL, diffuse large B-cell lymphoma; BENTA, B-cell expansion with NF-κB and T-cell anergy; MCL, mantle cell lymphoma; CLL, chronic lymphocytic/leukemia.

A deregulation of MALT1 function is associated with the development of various diseases. Patients with inactivating MALT1 mutations develop immunodeficiency (91–93) or immune dysregulation, polyendocrinopathy, enteropathy, and X-linked (IPEX)-like syndrome (94). Patients with an oncogenic activation of the MALT1 signaling pathway, on the other hand, develop lymphoid malignancies or lymphoproliferative disease (Figures 1,4) (95, 96). An increasing number of T and B cell malignancies are now recognized to be driven by constitutive antigen receptor signaling as a result of foreign or self-antigen recognition (97) and/or gain-of function mutations (Figures 4A,B) that trigger CBM complex formation and downstream signaling (95, 98–104). Such mutations can be found in the CBM component CARMA1 itself (98) or in the upstream components of the pathway, including the B-cell receptor-associated CD79 chains (103), the T-cell coreceptor CD28, PLCγ1 and PKCβ (95). Lymphomas with such mutations include the activated B-cell (ABC) subtype of diffuse large B-cell lymphoma (DLBCL) (98, 99, 103, 105, 106), in which chronic active BCR signaling is often combined with oncogenic activation of the TLR signaling component MyD88 (107). Constitutive CBM-dependent signaling is also found in mantle cell lymphomas (MCL) (104, 108, 109), chronic lymphocytic leukemia (CLL) (110), acute T-cell leukemia/lymphoma (ATLL) (102), cutaneous T-cell lymphoma (CTCL) and Sézary syndrome (100, 101), as well as peripheral T-cell lymphomas (PTCL) (111) (Figures 4A,B). The relevance of the CBM complex and MALT1 protease activity were formally shown by silencing of the individual CBM components in ABC DLBCL (112, 113) and MCL (104) cell lines, or by inhibition of MALT1 proteolytic function in ABC DLBCL (113, 114), MCL (104), and CLL (110) cell lines. Interestingly, gain of function mutations of CARMA1 were also reported in patients with a congenital lymphoproliferative disease known as BENTA (B-cell expansion with NF-κB and T-cell anergy) syndrome (115–117) (Figures 4A,B).

In some types of lymphomas, constitutive MALT1 activation is achieved by mechanisms that bypass the classical CBM signaling events. These include chromosomal translocations involving the MALT1 or BCL10 genes, which are found in MALT lymphomas (1). Such translocations can lead to overexpression of the BCL10 or MALT1 genes or, more frequently, to the generation of an oncogenic API2-MALT1 fusion protein. This fusion protein activates the classical (canonical) and alternative (non-canonical) NF-κB pathways through its scaffold and protease function thereby bypassing the BCR-dependent upstream signaling events (1). The API2-MALT1 fusion protein has constitutive protease activity and cleaves NIK (76) and LIMA1α (75), two non-canonical MALT1 substrates recruited by the cIAP2 moiety (Figure 4C). NIK cleavage leads to stabilization of NIK, which promotes activation of the non-canonical NF-κB pathway by phosphorylation and proteolytic maturation of the NF-κB2 precursor p100 into a mature p52 NF-κB2 subunit (76). Cleavage of LIMA1α disrupts the tumor suppressor function of this protein and promotes B cell proliferation and adhesion via the cleavage of its Lim domain, an LMO-like oncogenic protein (75) (Figure 4C). The cleavage of NIK and LIMA1α seems to be a specific oncogenic feature of the cIAP2-MALT1 fusion protein since it is not known to be triggered by antigen receptor-mediated MALT1 activation.

Recently, we have uncovered a role for MALT1 protease activity in the development of primary effusion lymphoma (PEL) (118). PEL is a lymphoid tumor triggered by Kaposi's sarcoma herpes virus (KSHV), an oncogenic virus also known as human herpes virus-8 (HHV8). KSHV-derived tumors derive from latently infected B cells that are characterized by constitutive NF-κB activation, which suppress the lytic program of the virus. The latency program is controlled by different viral latency genes such as K13 and K15, encoding a viral FLIP (Flice-like inhibitory protein) homolog and a membrane protein homologous to the latent membrane protein 2A (LMP2A), respectively. K13 and K15 induce constitutive activation of NF-κB via the protease and scaffold activity of MALT1 (118) (Figure 4D). Pharmacological inhibition or silencing of MALT1 leads to the reactivation of the viral lytic program and induces cell death of PEL cell lines *in vitro*. MALT1 inhibitors also efficiently block the development of PELs and induce their regression *in vivo*, in a xenograft model (118). Interestingly, K13 and K15 contribute to MALT1 activation in a CARMA1-independent manner (Figure 4D). Whether K13 and K15 mediate MALT1 activation via other CARMA/CARD proteins, or in an entirely distinct manner, remains to be explored.

## MALT1 deregulation is associated with allergy, inflammatory skin diseases and carcinoma

Deregulations of MALT1 function are not restricted to the above-described lymphoid pathologies but may also play wider roles in allergies, psoriasis, and various non-lymphoid cancers (Figure 1).

Evidence for a potential contribution of MALT1 to allergic asthma comes from two studies showing that CARMA1 is important in mouse models of allergic asthma (119, 120). CARMA3 has also been shown to play an important role in allergic airway inflammation in mice (121). The exact upstream events triggering CBM activation in these models remain undefined.

CARMA2/CARD14-mediated activation of MALT1 has been shown to play a major role in the activation of human keratinocytes upon exposure to the yeast cell wall component zymosan or *S. aureus* (15). Interestingly, activating mutations in the CARMA2-encoding gene CARD14 have been found to be associated with development of psoriasis, an inflammatory skin disease, in human patients (122, 123). Since then, an increasing number of studies have reported genetic mutations in CARD14 associated to psoriasis diseases (124). Some of the CARD14 mutations found in patients were shown to enhance NF-κB activation and promote secretion of CCL20 and IL-8, two chemokines known to be associated with psoriasis (123, 124). Similar to CARD11 mutations associated with lymphoid malignancies, CARD14 mutations were mostly found in the CC region. This suggests a similar mechanism of activation, via induction of an active CARD14 conformation and oligomerization necessary for CBM complex formation and downstream signaling events. In support of this model, a recent study showed that a single point mutation in CARD14 E138A, which renders CARD14 hyperactive, was sufficient to drive spontaneous psoriasis disease in mice (125). Inhibition of MALT1 protease activity effectively reduces cytokine and chemokine expression induced by overexpression of this hyperactive CARD14 mutant in human primary keratinocytes (126). Therefore, topical application of MALT1 inhibitors may be a future treatment option for psoriasis patients.

Finally, an increasing number of publications support a role for aberrant MALT1 signaling in the development of non-lymphoid cancers, such as glioblastoma (127), breast cancer (18, 128, 129), melanoma (130), lung cancer (18), and various other carcinomas (1, 131, 132). In these cancers, MALT1 seems to be activated by constitutive signaling from GPCRs or the EGFR via CARMA3 and/or by overexpression of MALT1 through various mechanisms.

For breast cancer, two different subtypes, which are HER2 or angiotensin II receptor (AGTR1) positive, have been described to be addicted to CBM signaling via CARMA3 (18, 128, 129). Interestingly, these two subsets show abnormal expressions of AGTR1 or HER2 in a mutually exclusive manner (128). In AGTR1 and HER2 positive breast cancer cell lines, NF-κB is activated upon stimulation with Angiotensin II or the HER2 ligand heregulin, respectively, in a CARMA3-dependent manner (18, 128, 129). Silencing of CBM components abrogates tumor cell growth, migration, and invasion (18, 128, 129) and affects the tumor microenvironment, since endothelial cell chemotaxis and angiogenesis are abolished (128). AGTR1-positive breast cancers represent 15–20% of all breast cancers (128) and HER2 positive breast cancers form 20–25% of all breast cancers (18, 128, 129, 133). It remains possible that other breast cancer subtypes depend on constitutive signaling by other GPCRs that can trigger NF-κB signaling via CARMA3 (134). In addition to its relevance in breast cancer, CARMA3 has been shown to play a role in lung cancer (18). In this context, EGFR signaling via CARMA3, BCL10, and MALT1 promotes tumorigenesis through effects on NF-κB activation, cell proliferation, migration and invasion. The relevance of the proteolytic activity of MALT1 was not formally assessed in the above-cited models of breast cancer (128) and lung cancer (18). It would thus be interesting to test the potential effectiveness of pharmacological MALT1 inhibitors in these models.

The relevance of MALT1 as a protooncogene has been recently extended to additional malignancies including melanoma (130) and glioblastoma (127), in which MALT1 can be overexpressed. Indeed, MALT1 is highly expressed in a subset of melanoma associated with poor prognosis (130). MALT1 downregulation or inhibition with the MALT1 inhibitor MI-2 (135), diminishes the growth and the dissemination of melanoma cells in the lung (130). While the proliferation of these melanoma cells is NF-κB dependent, the dissemination is JNK/c-Jun-dependent (130). In glioblastoma, it was shown that a subset of tumors with poor outcome expresses low levels of the microRNA miR181d, leading to high NF-κB activation. One of the targets of miR181d is MALT1, which is highly expressed in the miR181d-low tumors (127). Ectopic expression of miR181d and the consequent downregulation of MALT1 suppresses growth of tumor cells (127). These two studies suggest that, like in a subset of MALT lymphomas (136), MALT1 overexpression *per se* may play a role in promoting carcinogenesis, potentially by spontaneous aggregation-mediated activation of MALT1. Future studies should address how MALT1 promotes cellular transformation in various cancers, whether and how specific substrates of MALT1 contribute to tumorigenesis and if MALT1 inhibitors might be of therapeutic interest.

## MALT1 inhibitors may be of therapeutic interest for various pathologies

Several recent studies have reported the development of small molecule MALT1 inhibitors that act as active site or allosteric inhibitors (135, 137–140). These have therapeutic effects in preclinical (mouse) models of diffuse large B-cell lymphoma, (135, 137, 138) and primary effusion lymphoma (118). The idea that MALT1 inhibitors may be useful to dampen inflammatory immune responses has received support from studies using a mouse model of multiple sclerosis, in which paralysis symptoms are alleviated by treatment with a MALT1 inhibitor, or by genetic inactivation of MALT1 (13, 65, 85). The latter also protects mice from experimental induction of colitis (13). Moreover, pharmacological inhibition of MALT1 protects mice from LPS-induced inflammation and lung injury, most likely through inhibitory effects on myeloid cells (141). Nevertheless, the assumption that MALT1 inhibitors will generally dampen immune responses has been challenged by the observation that mice expressing a catalytically inactive form of MALT1 have reduced numbers of natural Treg cells and, as a consequence, progressively develop inflammatory autoimmune disease (13, 14, 65, 66). This raises the interesting possibility that, at least in the context of cancer, MALT1 inhibitors may exert their effects by a dual action on both the inhibition of the growth of cancer cells and boosting anti-tumor immune responses. Another possible field of application for MALT1 inhibitors are inflammatory skin diseases such as psoriasis, in which inhibitory effects on keratinocytes and possibly on skin T cells may benefit the patients (15, 124–126). As we progress in our knowledge about the molecular and biological function of MALT1, future applications are likely to emerge.

## Author contributions

All authors listed have made a substantial, direct and intellectual contribution to the work, and approved it for publication.

### Conflict of interest statement

The authors declare that the research was conducted in the absence of any commercial or financial relationships that could be construed as a potential conflict of interest.

## Abbreviations

ABC: activated B-cell
AGTR1: angiotensin II receptor
ATLL: adult T-cell leukemia/lymphoma
BCL10: B-cell lymphoma-10
BCR: B-cell receptor
BENTA: B-cell expansion with NF-κB and T-cell anergy
CARD: caspase recruitment domain
CARMA: CARD-containing membrane-associated guanylate kinase
CBM: CARMA/CARD-BCL10-MALT1
CC: coil-coiled
CDE: constitutive decay element
CLL: chronic lymphocytic leukemia
CTCL: cutaneous T-cell lymphoma
DD: death domain
DC: dentritic cells
DLBCL: diffuse large B-cell lymphoma
DUB: deubiquitinating enzyme
EGFR: epidermal growth factor receptor
FcR: Fc receptor
FLIP: Flice-like inhibitory protein
GPCR: G protein-coupled receptor
GUK: guanylate kinase
HER2: human EGFR-2
HOIL1: heme-oxidized IRP2 ubiquitin ligase 1
HOIP: HOIL1-interacting protein
HHV8: human herpes virus-8
Ig: Immunoglobulin
IPEX: immune dysregulation, polyendocrinopathy, enteropathy, X-linked
ITAM: immunoreceptor tyrosine-based activation motif
KSHV: Kaposi's sarcoma-associated herpes virus
LMP2A: latent membrane protein 2A
LUBAC: linear ubiquitin chain assembly complex
Mφ: macrophage
MAGUK: membrane-associated guanylate kinase
MALT: mucosa-associated lymphoid tissue
MC: myeloid cell
MCL: mantle cell lymphoma
mTORc1: mammalian target of rapamycin complex 1
MZ: marginal zone
nTreg: natural Treg
JNK: c-Jun N-terminal kinase
NF-κB: nuclear factor kappa B
NK: natural killer
OSCAR: osteoclast associated receptor
PDZ: PSD95 Dlg1 ZO-1
PEL: primary effusion lymphoma
PI: protease inactive
PKC: protein kinase C
PTCL: peripheral T-cell lymphoma
RTK: receptor tyrosine kinases
TCR: T cell receptor
TREM-1: triggering expression by myeloid cells 1
PTM: posttranslational modification
RA: rheumatoid arthritis
SH3: Src homology 3
SHARPIN: SHANK-associated RH domain interacting protein
UTR: untranslated region.
